# From Classical to Advanced Use of Polymers in Food and Beverage Applications

**DOI:** 10.3390/polym14224954

**Published:** 2022-11-16

**Authors:** Saúl Vallejos, Miriam Trigo-López, Ana Arnaiz, Álvaro Miguel, Asunción Muñoz, Aránzazu Mendía, José Miguel García

**Affiliations:** 1 Departamento de Química, Facultad de Ciencias, Universidad de Burgos, Plaza de Misael Bañuelos s/n, 09001 Burgos, Spain; 2 Centro de Biotecnología y Genómica de Plantas, Instituto Nacional de Investigación y Tecnología Agraria y Alimentaria, Campus de Montegancedo, Universidad Politécnica de Madrid (UPM), 28223 Madrid, Spain; 3 Facultad de Ciencias, Campus de Cantoblanco, Universidad Autónoma de Madrid, Calle Francisco Tomás y Valiente 7, 28049 Madrid, Spain

**Keywords:** advanced food packaging, polymer sensors, active packaging, water treatment, separation of target molecules

## Abstract

Polymers are extensively used in food and beverage packaging to shield against contaminants and external damage due to their barrier properties, protecting the goods inside and reducing waste. However, current trends in polymers for food, water, and beverage applications are moving forward into the design and preparation of advanced polymers, which can act as active packaging, bearing active ingredients in their formulation, or controlling the head-space composition to extend the shelf-life of the goods inside. In addition, polymers can serve as sensory polymers to detect and indicate the presence of target species, including contaminants of food quality indicators, or even to remove or separate target species for later quantification. Polymers are nowadays essential materials for both food safety and the extension of food shelf-life, which are key goals of the food industry, and the irruption of smart materials is opening new opportunities for going even further in these goals. This review describes the state of the art following the last 10 years of research within the field of food and beverage polymer’s applications, covering present applications, perspectives, and concerns related to waste generation and the circular economy.

## 1. Introduction

Polymer science and technology is devoted to the development of materials with unique functionalities for packaging, construction, medical applications, electronics, or aeronautics due to their tunable nature and a wide variety of physical and chemical properties. Since their industrial development in the 1930s, polymers have been and will be a fundamental pillar of the food industry as we know it nowadays. The transport, storage, and conservation of practically all food products at a reasonable price is only possible due to a perfect balance between the mechanical properties of polymers, their price, low weight, and barrier effect against different gases. Traditional polymers used in food packaging applications include polyethylene (PE), polyethylene terephthalate (PET), or polystyrene (PS), among others. They serve as protection against chemical, biological, and physical damage and prevent the loss of aroma, flavor, and/or antioxidants. They ensure an adequate balance of gases and humidity inside the packaged food, increasing the shelf life of food and facilitating its handling [1,2,3].

Current trends in food packaging go a step forward to serve as active packaging too, delivering chemical species such as antioxidants and antimicrobials in their polymer formulation that are released (or not) from the package to protect the goods inside and extend their shelf-life. Also, the use of edible polymers or biopolymers for food packaging is gaining interest for reducing waste. In addition to protecting the edible goods inside any package, researchers in polymers for food and beverage applications have leapt to develop polymers for advanced applications [4,5,6,7,8,9,10,11,12,13,14,15]. Such innovations are smart polymers for the detection of targets (chemical species, physical stimuli, microorganisms, etc.), extraction of contaminants, separation of compounds of interest, controlled release of species in active packaging, etc.

Polymers play a critical role in the food industry in many different senses. This work aims to perform a multi-view review to classify the different characteristics and uses of polymers in this field for diverse profile readers. Firstly, a classification by applications in which the study of relevant contributions based on advanced polymers for food packaging, target species detection and authentication, water and beverage treatment, and the separation of compounds has been carried out. Secondly, a classification of polymers widely used in food packaging is presented, with a discussion of the relationship between their chemical structures and their properties. Finally, classification by environmental concerns was conducted, since “plastics pollution” is one of the significant concerns and one of the great challenges facing contemporary society.

## 2. Classified by Application (Advanced Polymers)

**Figure 1:**
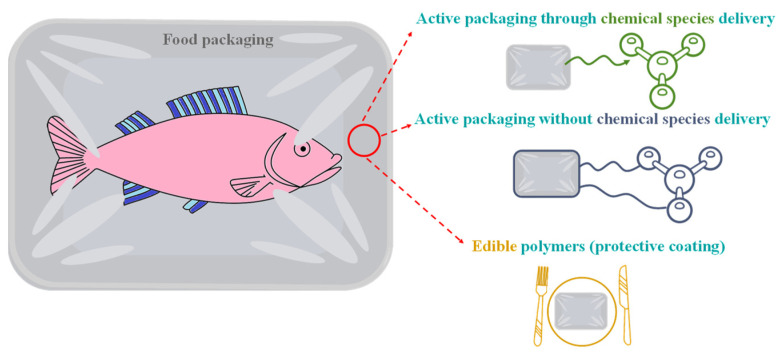
Classification for different food packaging applications, namely, active packaging through/without chemical species delivery and edible polymers.

As mentioned, the main application of polymers in the food industry is food packaging. However, given the abundant literature in this area, we only focus on advanced food packaging, including active packaging within food packaging, differentiating between three types as graphically depicted in Figure 1:

- Those based on the migration of an active substance to perform a specific function (antimicrobial, antioxidant function, etc.)
- Those that are not based on the migration of a substance
- Edible and biopolymers

**Figure 2:**
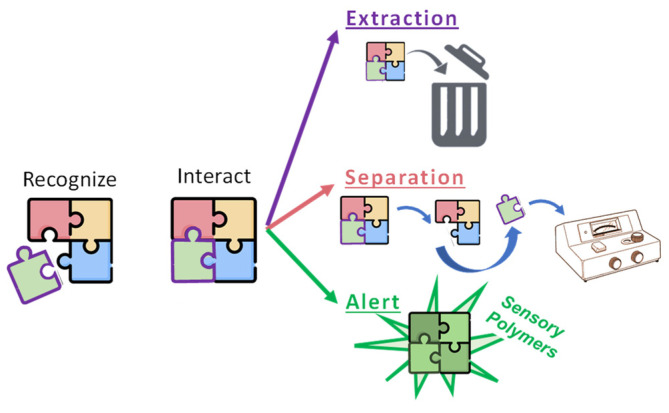
Differences between sensory polymers, polymers for extraction and elimination, and polymers for extraction and separation.

However, polymers in food applications can also be used to detect and/or quantify the presence of target species (sensory polymers), remove, or both determine and separate target species. Sometimes, it can be challenging to differentiate between these last applications since they all involve interaction with a target species. Though their final objective is different, they are defined schematically in Figure 2.

The classification categories depicted in Figure 2 have in common the need for a recognition mechanism, and the differences are herein described:

- Extraction and elimination of target species. The target is a dangerous species for the environment (pollutant, toxic substance, etc.) or to human beings (allergen, etc.). The main objective is to remove as much of this target as possible from the food or beverage. Although the polymer used for this purpose is sometimes recovered, the recovery of the target is not essential and is usually discarded.
- Extraction and separation of target species. The idea is the same as in the previous case, but in this section, recovering the target and determining its concentration is also part of the main objective. Outer equipment is used for measuring the concentration of the target (i.e., high-performance liquid chromatography, gas chromatography, spectrophotometry, etc.). The polymer is not involved in the quantification mechanism, which is independent of the polymer’s chemical structure.
- Alert, sensory polymers. The interaction between the target species and polymer produces a measurable signal (change in color, shape, hydrophilicity, conductivity, etc.). External measurement equipment may be used to record this signal, but unlike in the previous case, it is related to the polymer composition or chemical structure.

### 2.1. Advanced Food Packaging

Food packaging is the main and best-known application of polymeric materials in the food industry. The global production of plastics is distributed mainly in packaging (42%), automotive (8%), construction (20%), and the domestic environment (30%). Polymers have made significant advances in terms of quality and food safety [16].

Food packaging has grown from a mere food protection objective during manipulation to advanced functionalities aiming to extend their shelf-life and improve the attractiveness of the goods for consumption. The food packaging industry has progressed from using single-layer films to very complex multilayer films made from different polymers prepared by the co-extrusion or lamination of up to 13 layers or even more. The range of polymers used for food packaging is not very extensive, being the most common PE (low-density polyethylene, LDPE; high-density polyethylene, HDPE; linear low-density polyethylene LLDPE, polypropylene (PP) polyamide (PA; PA6, PA66), PET, PS, polyvinyl alcohol (EVOH), polyvinylidene chloride (PVDC), polyvinyl acetate (PVAc), poly(ethylene-co-vinyl acetate) (EVA), polycarbonate (PC), polyvinyl chloride (PVC), and poly(ethylene-co-acrylic acid) (EAA). Among them, the most extensively used are PE, PP, PA, PS, PET, and PVC. The characteristics and properties of single-layer films for packaging were described in scientific literature a long time ago. Despite not showing a wide variety, their combination into three or more layers, with the possibility of being repeated and the diversity of their fabrication methods, their design, and improvement are very complex. Each polymer has a function in a multilayer film, including gas/aroma barrier, scratch barrier, moisture barrier, mechanical and/or heat resistance, adhesiveness, printability, etc. On the other hand, the properties of multilayer films are a sum of the properties of the single components plus the synergic effects associated with the adhesiveness, the interfaces, and the addition of charges. However, despite the existing theoretical knowledge related to the materials’ properties, the films’ composition and the number and nature of the multilayers are just part of the know-how of the enterprises manufacturing them [6,15,17,18,19].

For this reason, in this review’s section, we only analyze relevant publications related to advanced packaging, including active packaging, i.e., packaging containing extra functionalities in addition to those related to protection from the environment. Otherwise, for general and technical information about the types of polymers used in food packaging, we remit the reader to Section 3 of this review.

#### 2.1.1. Active Packaging through Chemical Species Release

The main goal of active packaging is to control the package headspace composition during the shelf life of the goods inside to reduce food waste. Active packaging is not a new concept (the first patents date back to the beginning of the 20th century). Still, the controlled release of substances to the media or the absorption of others to preserve food means a new generation of packaging. In fact, it is a very extensive field since different ingredients show different preserving mechanisms. Most of these mechanisms go through the improvement of antioxidant and antimicrobial activities. Using active packaging instead of directly adding the substances to the bulk of the food can diminish the amount of substance required, especially through a controlled release, since the degradation or bacterial growth of food takes place mainly on the surface. However, the complex structure of food can vary the releasing or absorption rates and thus the efficiency and activity of the packaging, limiting the widespread use of active packaging [20].

Many different polymers are used within this frame, like natural polymers such as gelatin, starch, and chitosan, or synthetic polymers, such as poly(lactic acid) (PLA) or PP, which are the most relevant ones [21]. Table 1 summarizes relevant publications from the last 10 years regarding active ingredient release. The released species are widely known to act as antioxidants and antimicrobial agents, and they include silver and titanium nanoparticles, essential oils, tocopherol, nisin, carvacrol, thymol, anthocyanins, limonene, lignin, etc.

#### 2.1.2. Active Packaging without Chemical Species Release

Unlike active packaging with substances release, intrinsically antimicrobial polymers include the active ingredient in their chemical structure, showing essential advantages compared to the former: there is no migration of substances, and, therefore, the antimicrobial effect of the material is non-perishable, allowing the material to be reused. There are hardly any studies of intrinsically antimicrobial materials that are not based on the migration of a component whose bacterial inhibition values are reasonable and have been tested under real conditions.

González-Ceballos et al. used a copolymer of *N*-vinylpyrrolidone, methylmethacrylate, and a monomer with a covalently anchored vanillin derivative to prepare an antimicrobial absorbent food pad without the migration/delivery of substances [37]. The film-shaped pad was simply washable and reusable ten times at least, showing a shelf-life extension = 50%. The pads were tested in pork samples for *E. coli*, *S. aureus*, and *L. monocytogenes*, with inhibition up to 99.95%, 99.96%, and 99.02%, respectively. Delezuk et al. followed a different strategy. They used chitosan as the polymer, designing a micromotor-based bacteria-killing approach, relying on the combination of the inherent antimicrobial capacity of chitosan with the effective water-powered propulsion of magnesium micromotors, and showing a bacteria-killing efficiency of 96% within 10 min [38].

Furthermore, adding inexpensive fillers to the PLA matrix can lower costs, according to Spiridon et al. They studied formulations of PLA containing grape wastes and celery fibers to prepare composites with good mechanical and thermal properties and antimicrobial activity, appropriate for food-active packaging polymers, particularly when using grape waste [39].

#### 2.1.3. Edible Polymers and Biopolymers

Edible polymers are biodegradable and biocompatible polymer formulations applied on food surfaces as films that prevent food deterioration by providing barrier properties and enhancing quality and safety [40]. The major difference between edible polymers and others used for food packaging is that the former becomes part of the food while the latter becomes waste. Edible polymers are generally applied as a coating on certain foods, but they can also be prepared as film-shaped materials and serve as packaging. These polymers improve and preserve food quality and can be both classic synthetic polymers and biopolymers. Still, the latter has more relevance nowadays since they are usually environmentally friendly, that is, biodegradable polymers obtained from natural resources (green polymers) [41,42].

The use of these polymers in no case can compromise the quality of food, and their functions range from inhibiting lipid oxidation to diminishing metmyoglobin development in fresh meat. Edible films prepared from milk proteins (such as casein, lactoferrin, or whey protein) have proven to be a reliable and firm alternative to polymers derived from oil and used in food packaging [43,44]. Furthermore, these polymers can also be used for the controlled delivery of different substances, such as preservatives, provided that they are included in the list of products accepted as food-grade [41]. Edible coatings, and especially those made with biopolymers, are a sustainable alternative that offers a wide range of possibilities in terms of functionality, such as barrier properties (low permeability to water vapor and oxygen) for totally protective food packaging [45].

Over the last few years, academic research has given relevant importance to polysaccharides for the manufacture of edible polymers and biopolymers for food packaging, which seems quite logical since they are biodegradable, not toxic, can be manufactured as films, are widely available in nature, and have good compatibility with cellulose-based supports [18]. However, they are polymers that are quite sensitive to humidity, which limits their use on a large-scale basis, as the main drawback. On the other hand, this also opens the door to new lines of research aimed at the chemical modification of polysaccharides and other natural polymers, introducing different additives in their formulations for improving mechanical and thermal properties, decreasing the permeability to water vapor, and increasing elongation at break, tensile, and antimicrobial properties [45,46].

Some of the properties of edible polymers can also be enhanced when combined with natural antimicrobial compounds. Piñeros-Hernandez et al. used polyphenol-rich rosemary extracts to introduce them into cassava starch films to prepare active food packaging with antioxidant capacity. The polyphenol content ranged from 4.4 to 13.6 mg of gallic acid equivalents per gram. In addition to enhancing the materials’ antioxidant properties, other properties such as barrier protection against ultraviolet light, mechanical properties, permeability to water vapor, or hydrophobicity vary with the amount of rosemary extract added to the films [47]. In another example, Elsabee et al. described different strategies to improve the mechanical properties of an edible polymer, such as chitosan, a marine-available, biocompatible, biodegradable polysaccharide with antibacterial and antifungal properties. Some of these strategies are based on adding to chitosan other natural polymers, such as starch, essential oils, or clays [48].

Davachi et al. describe the natural polymer salvia macrosiphon seed mucilage and the enhancement of target properties by adding small amounts (≈2%) of glycerol and nanoclays [46]. Jang et al. prepared edible polymers with high tensile strength using the residues generated in the rapeseed oil extraction process. These residues are rich in a major protein comprised of two polypeptide chains, α, and β, of 20 kDa and 30 kDa, linked by a disulfide bond. The film-forming solution was prepared with the residues, plasticizers (sorbitol and sucrose), emulsifiers (polysorbate), and gelatin (*gelidium corneum*) [49].

### 2.2. Target Species Detection and Quantification (Sensory Polymers)

Food control and safety are among the most critical areas in the food industry. In this framework, polymers, specifically smart polymers, have been a great revolution in the last decade because of their low costs, ease of use, and versatility in adapting to any target species [19]. A smart polymer is defined as a polymer that, through a specific mechanism, offers a response to a specific stimulus. Therefore, these polymers can be classified by the stimulus (chemical or physical), by the signal transduction mechanism (conducting polymers, molecularly imprinted polymers (MIPs), dosimeters, indicator displacement assays or IDAs, etc.), or by the response (colorimetric, fluorometric, electrical, etc.). However, a key concept defines sensory polymers: the response is an alert. In other words, smart polymers are divided into responsive polymers or sensory polymers if they respond with an action (drug delivery, change in size or shape, etc.) or with an alert, respectively [50].

Among the sensory polymers in food applications, the most relevant are classified based on the following targets: drugs, smell and taste, biogenic amines, heavy metals, humidity and gases, temperature and pH, nitrates and nitrites, and microorganisms, among others.

#### 2.2.1. Drugs

Human beings release different drugs into the environment through the biological waste generated or through a deficient wastewater purification process. These compounds are not adsorbed in the subsoil and end up in underground aquifers. Therefore, they pose a serious environmental problem that has aroused scientific interest in different fields, especially in drinking-water control and monitoring.

**Figure 3:**
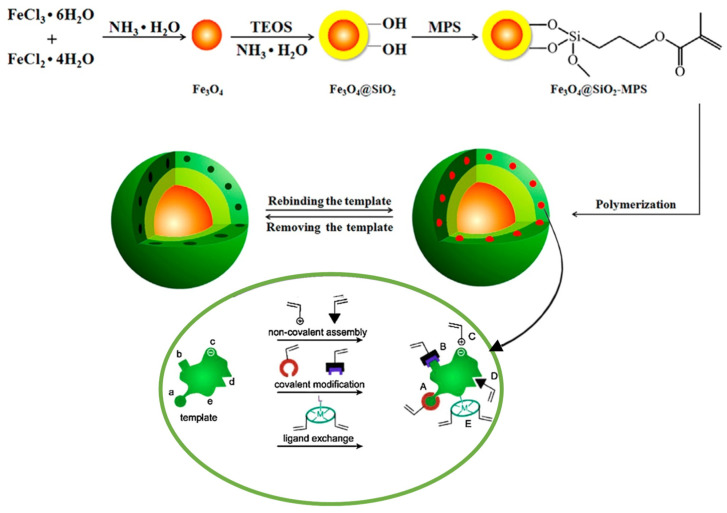
Schematic description of the synthesis and preparation of a magnetic MIP. The procedure goes through the obtention of iron (II, II) oxide (magnetite) from the co-precipitation of FeCl·H_2_O and FeCl_3_·6H_2_O in NaOH solution or ammonia. Then the magnetite surface is modified by silanization using TEOS (tetraethyl orthosilicate) and functionalized using MPS or other silanes with multiple bonds for further polymerization. The MIP is then prepared using different crosslinkers and monomers. The last stage consists of removing the template molecules after polymerization. Adapted with permission from [54]. Copyright © Elsevier 2017.

Undeniably, antibiotics are one of the most sought-after and controlled targets. Xiao et al. designed 3D polymer slides based on polylysine to detect cephalosporins, aminoglycosides, and sulfonamide antibiotics in pork and milk. However, the detection of antibiotics has also attracted the scientific community’s attention. Madikizela et al. describe a MIP for the solid-phase detection of nonsteroidal anti-inflammatory drugs such as naproxen, ibuprofen, and diclofenac [51]. MIPs are synthetic polymers with unique molecular recognition capabilities showing high selectivity for target species by mimicking the interactions taking place in natural receptors (such as antigen–antibody) but without stability restrictions. The detection system based on MIPs is complemented by a high-resolution liquid chromatograph equipped with photodiode array detection. As a result, limits of detection of 0.15, 1.00, and 0.63 μg L^−1^ were reached for naproxen, ibuprofen, and diclofenac, respectively, and the most abundant drug of all in the different analyzed samples was ibuprofen, sometimes reaching concentrations higher than 220 μg L^−1^. In fact, MIPs are one of the most broadly used polymers in this section, and other authors have described sensory polymers for the detection of tetracycline drugs in animal-derived foods [52] or the determination of streptomycin residues in food [53], among others [54]. A schematic representation of a magnetic MIP synthesis and preparation for the determination of drugs is described in Figure 3.

Another type of polymer chosen by many authors to prepare sensory polymers for drug detection are conductive polymers. Karaseva et al. designed a sensory polymer based on polypyrrole to detect trace quantities of chloramphenicol, a veterinary drug for treating and preventing infectious diseases [55]. Similar systems can be found in the literature for 6-mercaptopurine detection (an anticancer drug used to treat leukemia) or piroxicam detection (a nonsteroidal anti-inflammatory drug) [56].

#### 2.2.2. Smell and Taste 

In industrial sectors, such as the food or even the automotive industry, aromas are evaluated by a group of skilled panelists through sensory analysis. The human nose and tongue can recognize millions of odors/tastes with a precision that no other analytical instrument has been able to reach up to date. 

However, these analyses are always associated with human errors, such as individual variability. Electronic nose systems (e-nose) are a suitable alternative for scent recognition. These systems are made up of one or several chemical sensors capable of reacting with different gases and emitting a measurable signal. These systems produce a huge amount of data, so they are usually complemented with advanced mathematical procedures for data processing [57].

For this reason, the efforts of the scientific community to create e-noses and tongues are enormous since objectively determining tastes instead of using subjective sensory assessment and developing novel synthetic flavors in the food and beverage industry can be an advantageous methodology. Furthermore, polymers play a relevant role since the choice of the type of polymer and the method to integrate them into the systems (deposition, coating, etc.) directly affect the sensor’s performance. Thus, many authors have generated excellent results over the last decade, as summarized herein.

Ahn et al. have developed an e-tongue for umami and sweet taste based on nanovesicles immobilized on poly(D-lysine) [58]. Related to milk analysis, Tazi et al. developed an e-tongue made of PVC but comprising a matrix of 16 different lipid/polymer membranes and performed a proof of concept by monitoring the flavor evolution in milk samples [59]. Additionally, Pérez-González et al. preferred to rely on PVC for the preparation of their e-tongue, which detected various compounds typically present in milk, including salts (CaCl_2_, KCl, and NaCl), sugars (galactose, glucose, and lactose), lactic acid, and organic acids such as citric acid [60].

This approach has also been applied to fruits, such as oranges. Gruber et al. designed an e-tongue based on four conducting polymers, poly(9,9-dioctyl-2,7-fluorenyleneethylene), poly(2,5-biphenyleneethylene), poly(4′-hexyloxy-2,5-biphenyleneethylene), and poly (2-bromo-5-hexyloxy-1,4-phenylenevinylene). The device can detect *Penicillium digitatum*, an important pest in all citrus-producing countries [61].

Some food additives, such as sugar, have also been the subject of research with e-noses. Péres et al. designed a low-cost chemoresistive gas sensor from a thin film of poly(2-dodecanoylsulfanyl-*p*-phenylenevinylene), a conductive polymer doped with dodecylbenzenesulfonic acid, and applied their development to the determination of methanol in sugar-cane spirit [62].

In another relevant investigation, Mahato et al. analyzed five bottled-water samples with three different e-tongues based on three functionalized polymer membrane electrodes: phosphorylated and crosslinked polyvinyl-co-ethylene membrane, phosphorylated hexadecyl trimethyl ammonium chloride modified EVOH-polyacrylic acid membrane and phosphorylated and crosslinked EVOH membrane. After analyzing the results, the authors concluded that the system could detect dissolved minerals in tested samples [63].

#### 2.2.3. Biogenic Amines 

Biogenic amines (BAs) are formed during the oxidative decarboxylation of the amino acids present in food caused by the breakup of proteins by microorganisms. Thus, they are generated in some foods’ aging or decomposition processes, mainly fish, although their detection in cheeses is also of great importance [19]. These organic compounds are well-established indicators of food freshness and can cause severe harm to organisms after excessive intake. Most BAs, except histamine, have high vapor pressure and can be detected in the gas phase. The most studied and abundant BAs in fish spoilage processes are tryptamine, histamine, trimethylamine, putrescine, 2-phenylethylamine, cadaverine, spermidine, spermine, tyramine, 1,7-diaminoheptane, and 1,6-diaminohexane.

In general, many of the investigations devoted to detecting the presence of BAs do not differentiate between specific amines since all kinds of BAs are formed in the deterioration of food. However, the BA detection mechanisms and the strategies followed are quite diverse. For example, for the detection of biogenic amines, Yuroba et al. prepared cellulose acetate electrospun nanofibers doped with the amine-reactive chameleon dye Py-1. Upon contact with BAs, the nanofiber mat converts the weak fluorescent pyrylium dye Py-1 into a pyridinium dye strongly emitting red [64]. Another strategy takes advantage of dopamine polymerization since this polymerization is very sensitive to BAs, indicating their presence [65].

**Figure 4:**
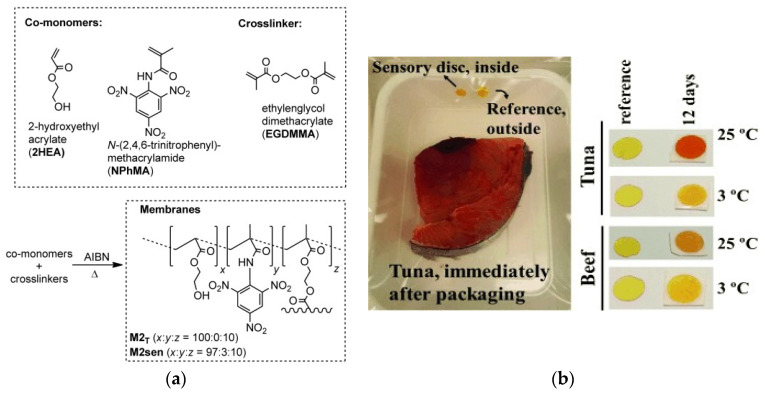
Determination of biogenic amines in tuna using acrylic polymers: (**a**) monomers and chemical structure of the sensory membrane and the (**b**) color development of the sensory polymers inside tray-packaged tuna under different ambient conditions. Adapted with permission from [67]. Copyright © John Wiley and Sons 2015.

On the other hand, Chow and coworkers prepared PS with 3 novel heterobimetallic Ru(II)–Ln(III) donor–acceptor complexes for the live control of BAs vapors. They applied this system to fish samples (Atlantic mackerel) by analyzing the spectrofluorimetric behavior [66]. The inclusion of sensory motifs in the polymer chain is another efficient strategy followed by some authors for the colorimetric or fluorometric detection of BAs. In this sense, Pablos et al. prepared a poly(2-hydroxyethyl methacrylate) with trinitrotoluene derivative motifs, able to react with amines to form the red-colored Meisenheimer complex (Figure 4) [67]. Furthermore, functional aromatic PAs containing pendant phthalimide derivatives were synthesized to react with BAs, causing a change in fluorescence and color, and the concentration can be measured using a smartphone [68].

Further, some examples of one single BA as the target species can be found in the literature. Vasconcelos et al. were able to detect putrescine using EVA containing maleic anhydride. Putrescine binds the maleic anhydride, triggering the resultant swelling of the polymer with spectral variations from an optical point of view [69]. Kumar and coworkers detected cadaverine, in addition to putrescine, with a poly(neutral red) electrochemical sensor. The positively charged groups on the polymer surface form ion-pair complexes with cadaverine or putrescine through diphosphate ion bridging, facilitating the detection and the formation of ion-pair [70]. Trimethylamine (TMA) was also solely detected using a copolymer of 2-hydroxyethyl methacrylate, 2-ethoxyethyl methacrylate, and an occluded pyrylium salt in the polymeric films, able to react with BAs, producing a unique change of color in the presence of TMA vapors [71].

As mentioned, histamine is the most challenging BA to be detected due to its low vapor pressure. However, Akhoundian et al. and Mattson et al. were able to detect it using MIP based on polymethacrylic acid incorporated into a carbon paste electrode [72,73].

#### 2.2.4. Heavy Metals

Heavy metals have always been one of the most investigated contaminants due to their presence in water and various foods and their high toxicity to humans and the environment. Among the heavy metals, mercury is undoubtedly one of the most relevant, although sensing metals, including arsenic, cadmium, nickel, or lead, are also of great importance [74].

These metals reach the aquifers and the oceans through the pollution generated by combustion engines, brake pads, and emissions from industry, among others. Once in the environment, they are assimilated and bioaccumulated by organisms and pass into the food chain, directly affecting humans, so many research articles can be found concerning the detection of metals in fish.

**Figure 5:**
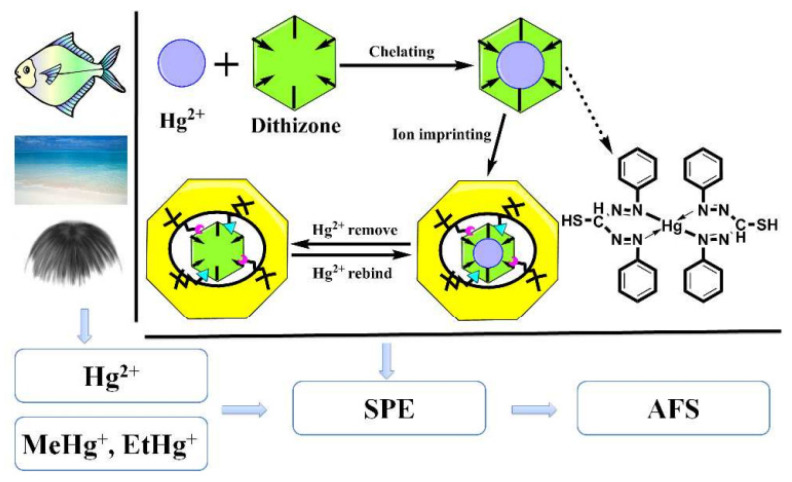
Schematic representation of the preparation of Hg-IIPs via the sol–gel process. The Hg(II) and dithizone complex was prepared in the first place and used as the template molecule. The template was pre-polymerized with functional APTES (a frequently used silane coupling agent in sol–gel processes). Hg(II) was eliminated by elution, leaving the specific recognition site. After Hg(II) or organic mercury is recognized by solid phase extraction (SPE), atomic fluorescence spectroscopy (AFS) is used for detection. Adapted with permission from [79]. Copyright © RSC Publishing 2011.

Some current strategies in the last 10 years use ion-imprinted polymers (IIPs) (an example is depicted in Figure 5) for the selective recognition of metal ions in water or food, reaching low limits of detection (0.03 ng mL^−1^). Nevertheless, other approaches rely on a color or fluorescent variation, the use of conducting polymers, or the polymerization mechanism in the presence of the metal ion, as summarized in Table 2.

**Table 2:** Classification of the most relevant works of the last ten years for the detection of heavy metals with sensory polymers, identifying the target, the medium, the type of polymer, and the interaction/response mechanism.

Target/Medium	Polymer	Comments	Ref.
Hg(II) and Pb(II) in water	Aptamer- functionalized colloidal photonic crystal hydrogel (CPCH) films of polyacrylamide	During detection and caused by the cross-linked aptamers, the hydrogel is shrunk as it binds heavy metal ions, resulting in a blue shift in the Bragg diffraction peak position of the CPCHs. The shift value serves to quantify the concentration of Hg(II) or Pb(II).	[75]
Hg(II) in water	Conjugated polymer synthesized based on fluorene and 1-CN	Based on the mercury(II) promoted deprotection reaction of dithioacetal; LOD = 1.0 × 10^−6^ mol L^−1^.	[76]
Mercury in 15 different fish	IIP based on *N*-(pyridin-2-ylmethyl)ethenamine coated on Fe_3_O_4_ nanoparticles	The developed sorbent was effectively applied to detect low amounts of Hg(II) ions in different fish samples (*Hydrocynus vittatus*, sardine, *Clarias mossambicus*, *Bagrus orientalis*, *Tilapia urolepis*, *Pseudotolithus*, *Selene dorsalis*, blue shark, *Alestes affinis*, meagre, *Hoplias malabaricus*, *Pagrus pagrus*, *Oreochromis niloticus*, *Bagre marinus*, and anchovy). LOD = 0.03 ng mL^−1^	[77]
Cd(II) and Pb(II) in fish samples	IIPs based on 2-(diethylamino) ethyl methacrylate and 8-hydroxyquinoline (complexating agent)	The polymer was used for a previous sample pre-concentration step, and the system was tested with samples from squid, horse mackerel, sardine, hake, grouper, and gilthead bream. LOD = 0.15 mg L^−1^ for Pb(II) and 0.50 mg L^−1^ for Cd(II)	[78]
Hg(II) and organic mercury in fish	IIPs based on poly(3-aminopropyltriethoxysilane)	The sensory systems can effectively cleanup, enrich, and determinate trace mercury species in complex matrices. LOD = 0.015 μg L^−1^ for Hg(II) and 0.02 μg L^−1^ for organic mercury	[79]
Hg(II) in fish	Nickel nanoparticles deposited on high-surface-area carbon porous materials (CPMs) around a triblock copolymer template	The nanoparticles were deposited on CPMs prepared using the direct template method on a triblock copolymer method following the self-assembly of phloroglucinol-formaldehyde resol. The system was effectively quantified Hg(II) in fish samples. LOD = 2.1 nM	[80]
Hg(II) and organic mercury in fish and drinking water	Polymeric film based on *N*-vinylpyrrolidone and methylmethacrylate containing covalently anchored dithizone motifs	Color variation from green to red allows for the detection of hake, swordfish, and salmon at the pbb level.	[81]
Hg(II) and organic mercury in fish	Poly(2-hydroxyethyl acrylate) modified with pendant fluorescent receptors	The sensory polymers suffer an OFF–ON fluorescence process in the presence of mercury and methyl mercury. The system was tested with swordfish, tuna, pangasius, conger, and dogfish. LOD = 6.6 × 10^−6^ M	[82]
Hg(II) in river water samples	Br-doped poly(3,4-ethylenedioxythiophene) (PEDOT) modified carbon paper	The system is based on an electrode with a narrower bandgap that reaches detection limits up to 0.3 nM.	[83]
Hg(II) in zebrafish and drinking water samples	Polymerization process of barbituric acid	Barbituric acid derivatives interact with Hg(II) and then deprotonate to render a polymer and precipitate. LOD = 9.0 × 10^−8^ M	[84]
Pb(II) in water and rice samples	IIP based on polymethacrylic acid	A selective system for quantifying selectively lead, in the presence of a number of interfering metals, was achieved using an IIP in the glassy carbon electrode. LOD = 0.01 μM	[85]
Zn(II) in pet food samples	Polymeric film based on *N*-vinylpyrrolidone and methylmethacrylate containing covalently anchored quinoline derivative motifs	Fluorescence variation in films with gel behavior (LOD = 29 μg/L; LOQ = 87 μg/L). The system was tested with 15 commercial pet foods.	[86]

#### 2.2.5. Temperature and pH 

Temperature sensors are very relevant in the food sector, especially in packaging. Keeping the cold chain is essential in transporting and storing refrigerated and frozen foods, such as fish, fruit, meat, etc. The great beneficiary of these sensors is always the final consumer, who receives information on the entire cold chain in the container, for example, with a colorimetric label. However, in general, neither the collecting/manufacturing companies, the transport companies, nor the markets are interested in this type of label since it brings to light errors in the food chain for which nobody wants to be held responsible. For this reason, temperature indicators are becoming more advanced and can indicate the exact moment when the cold chain was broken, namely, time–temperature indicators (TTIs). These systems are smart packaging devices that can monitor and reveal food quality loss through visualized information [87].

Sometimes, dual sensors are used to reach better systems, as in the study by Maddali et al., in which dual-mode temperature sensors are described [88]. The system is based on doped (oxidized) regiorandom poly(3-hexylthiophene), and a noticeable color variation from the doped state (blue) to the dedoped state (yellow) takes place when exposed to temperature. Furthermore, the colorimetric response can be complemented with the electrical signal, as doped films are electrically conductive. This combination leads to a sensory system with the broadest sensing range of any polymer-based temperature sensor described until today (from 30 to 75 °C). Although the authors did not perform a proof of concept with food, the temperature-sensing window aligns with food-packaging applications.

**Figure 6:**
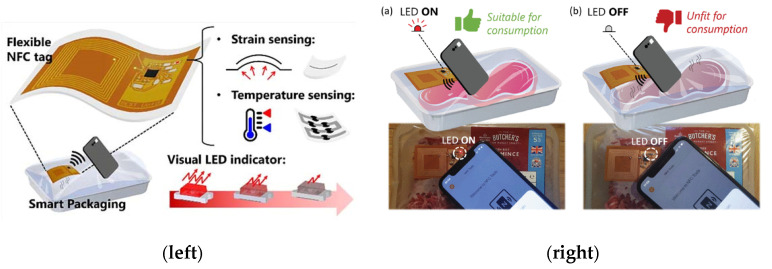
Smart tag with a near-field communication (NFC)-based sensing system to identify a dynamic strain or temperature visually semi-quantitatively: (**left**) the tag consists of a sensor (a microchannel-based sensor or a printed temperature sensor) and a LED as an indicator; (**right**) use of the sensor on a food package for meat spoilage determination causing blown pack spoilage. Figure by Pablo Escobedo et al. from [89], licensed under CC BY 4.0.

Similar systems with food-packaging potential applications have been described using other polymers, such as poly(3,4-ethylenedioxythiophene) polystyrene sulfonate (PEDOT:PSS) on a flexible PVC substrate (Figure 6a) [89], combinations of polydimethylsiloxane (PDMS) and EVOH [90], and polyimide [91]. An example is depicted in Figure 6b. Other authors have developed sensors for cryogenic temperatures (≈50K) based on poly(ethylene 2,6-naphthalate) [92] and sensors based on compostable and commercially available polymers [93]. 

The waste generated by food packaging is one of the significant concerns of contemporary society. For this reason, many authors have begun to base polymeric sensors for temperature control on compostable polymers. For example, Salvatore et al. used a compostable and commercially available polymer (Ecoflex^®^, by BASF) to prepare biodegradable and extremely deformable temperature sensors for the internet of things [93].

In addition to temperature control, pH control is highly relevant in the food industry, specifically in certain foods. When some pathogenic microorganisms multiply, there is a change in the pH of the medium. Therefore, the control and monitoring of the pH is a marker for the preparation of polymeric sensors indicating the real state of the food to the consumer to avoid possible intoxication. 

Some polymeric pH sensors are based on combinations of natural components such as *Euterpe oleracea* extract with polymers such as polycaprolactone (PCL) and polyethylene oxide [94]. Natural polymers are increasingly present in more products, and sensory polymers are no exception. For example, chitosan is not a polymer that responds to pH by itself, but it is an excellent support/matrix to house compounds such as phenol red or rosolic acid, providing materials with applications in food safety and biomedicine [95]. These types of sensors based on compounds or polymers of natural origin are the alternative to other polymeric sensors, such as those based on metal particles. For example, sensors prepared with polydiallyldimethylammonium [96] or polyimide [97], to which iridium oxide (IrO_X_) nanoparticles are added. 

However, only a few dual systems for pH and temperature (2 in 1) based on polymers and with proven applications in food safety have been reported. Among them, it is worth mentioning the study performed by Fucinõs et al. using poly(*N*-isopropylacrylamide) and poly(acrylic acid) [98] or the research by Topasna et al. using polyallylamine hydrochloride [99].

#### 2.2.6. Humidity, Gases, and Other Volatile Substances

The control of diverse volatile substances in food is especially relevant for food preservation. For example, humidity can cause various pathogens to proliferate, resulting in food spoilage. Therefore, different humidity control strategies were followed, including preparing materials with PVP combined with azo dyes, Disperse Red 1 (DR1), and 4-diethylamino-4′-nitrorazobenzene (DEA). This way, an easy and accessible way to produce humidity memory material that can be painted onto a variety of surfaces and shows a noticeable color change was enabled [100]. In another study, a conductive polymer (PEDOT:PPS) was applied to assess air quality, in the monitoring of the preservation state of foods, in the protection of walls, and even in wound-healing monitoring [101]. Similarly, for the control of humidity and aromas (apple, strawberry, and grape) added to gummy candies in the range of parts-per-billion, an e-nose based on polyaniline (PAni) was used, with LOD in the range of ppb [102].

Conductive polymers were also used to determine other volatile substances. Flexible films made of PAni were also used to rapidly detect ammonia as a spoilage indicator in protein-rich foods with limits of detection of around 50 ppm [103]. Another conductive polymer, PEDOT:PPS, in combination with functionalized multi-wall carbon nanotubes (CNTs), was used to determine formalin in squids. In the latter, electrons are transferred to the composites when formaldehyde molecules are adsorbed on PEDOT:PSS/CNTs’ surface. The hybrid PEDOT:PSS/CNTs’ gas sensors act as n-type nanomaterials experiencing a carrier concentration increase upon introducing electron-donating gases. This sensor reached a limit of detection (LOD) of 1 ppm [104].

#### 2.2.7. Nitrates and Nitrites 

Nitrates are related to diseases such as infantile methemoglobinemia, alterations in the central nervous system, and reduced motor activity. Furthermore, nitrates can be reduced in vivo to nitrites and react with amines to give rise to nitrosamines, highly carcinogenic compounds. The rise in nitrate and nitrite levels in drinking water is alarming, particularly in areas with agricultural and livestock farms. The use of inorganic and organic fertilizers in agricultural activity is the first source of contamination. However, it is also believed that manure and residues from livestock activity can contribute to the increase of the nitrates level in drinking water. Fertilizers can be a tremendous social and economic advantage, provided they are used responsibly. However, when used carelessly, the opposite effect occurs; that is, it causes an economic and environmental cost that must be assessed regarding its impact on water, climate change, ambient air quality, and waste generation [105,106]. Therefore, nitrate levels in drinking water should always be below 50 ppm. Levels between 50 and 100 ppm are acceptable but worrying, and levels above 100 ppm are hazardous [105].

For the detection of nitrates and nitrites, sensory polymers based on electrochemical responses are typically used. For example, Chu et al. developed a sensory polymer for nitrites based on three-dimensional copper nanodendrites electrodeposited on the surface of a poly(dimethyl diallyl ammonium chloride)-reduced graphene oxide modified glassy carbon electrode. The system reached a LOD of 0.06 μM and linear response from 1 to 15,000 μM. Similar systems have been reported based on classical PEDOT [107] but also on new polythiophene-derivative film-modified glassy carbon electrodes. The use of this new compound (2,5-di-thiophen-3-yl-thiazolo [5,4-d]thiazole) allowed the preparation of a nitrite sensor with a LOD of 2 nM, a linear response from 5.5 × 10^−9^ to 3.5 × 10^−5^ M, and excellent anti-interference ability. Another example was found using polypyronin on a pencil graphite electrode, which was tested in salami samples. The response of the sensory system was linear from 1.0 × 10^−6^ to 1.0 × 10^−4^ M, with a LOD = 5.0 × 10^−7^ M.

**Figure 7:**
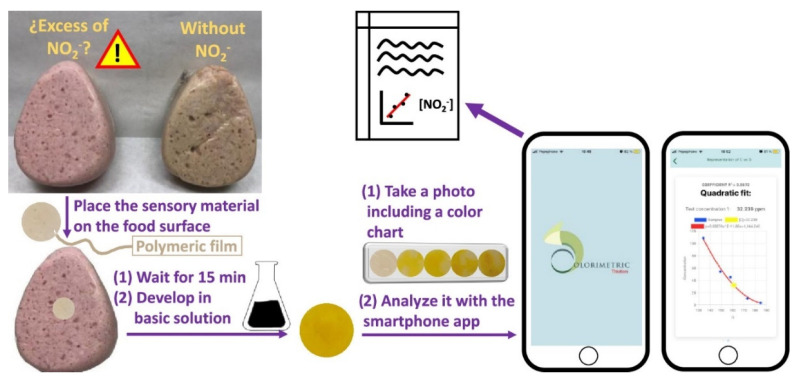
Measurement of the nitrite concentration in food samples using a sensory film-shaped polymeric film and analyzing the color change related to the amount of nitrite powered by the smartphone application Colorimetric Titration. Figure by Marta Guembe-García et al. from [109], licensed under CC BY 4.0.

Colorimetric and fluorometric sensors have also been designed to detect nitrate and nitrite anions. Pires et al. published a study about a new fluorometric nitrite biosensor with polythienothiophene–fullerene thin-film detectors for the control of water on-site, obtaining a LOD below 0.55 μM [108]. Nitrite detection is not only intended for drinking water, despite being the most relevant issue. The amount of nitrite added in processed meats should also be controlled since it is added to show a more pinkish color and a more appetizing appearance. González-Ceballos et al. recently published a work in which an amount of nitrite in meat was detected with a polymer sensor based on four monomers, *N*-vinylpyrrolidone, methyl methacrylate, a monomer containing an aromatic -NH_2_ group, and a monomer having an activated ring of phenol. The film-shaped polymers can change color by just contacting the material with the meat’s surface, and the analysis was boosted with a smartphone app specially designed for the objective. They carried out a proof of concept with more than 15 different meat products purchased in local supermarkets [109] (Figure 7).

#### 2.2.8. Microorganisms 

Identifying pathogenic microorganisms, mainly bacteria, in drinking water and food is relevant in key sectors of society, such as the medical field, food safety, and public health, which is why efficient and low-cost detection strategies are required [110]. 

**Figure 8:**
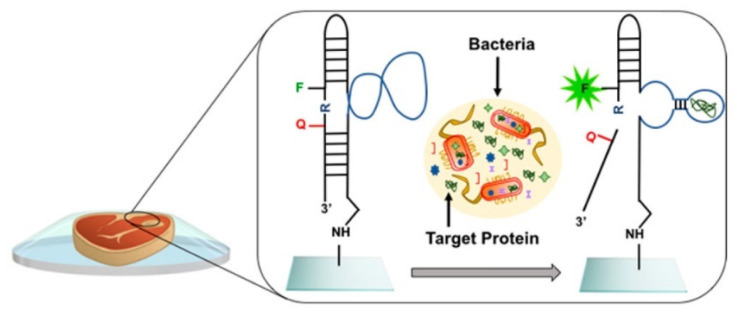
Representation of a sensitive DNAzyme sensor breaking up in the presence of *E. coli*. A transparent epoxy film supports a covalently bonded amine-terminated DNAzyme sensor. The connected fluorophor (F) and quencher (Q) are cleaved of the ribonucleotide in the presence of the target protein produced by *E. coli* cells. Adapted with permission from [111]. Copyright © American Chemical Society 2018.

In this sense, different detection systems for *E. coli*, a Gram-negative bacterium frequently found in the intestine of warm-blooded animals that can cause severe food poisoning, can be found in the literature. Yousefi et al. prepared cyclo-olefin polymer sensor films bearing epoxy functional groups (Figure 8). The system generates a fluorescence signal when *E. coli* is detected, and it was tested in meat and apple juice and worked at low concentrations, even at 10^3^ CFU/mL [111]. This microorganism was also detected in milk samples by a surface-imprinted polyurethane–urea sensor polymer for impedimetric measurements. The system showed an LOD of 120 CFU/mL [112] in water and milk samples using a “turn on” fluorescence sensor based on an amphiphilic conjugated polythiophene [113].

*S. aureus* is a bacteria found in the body’s microbiota in the skin and respiratory tract. However, it can become an opportunistic pathogen producing skin and respiratory diseases and food poisoning. To avoid this, Wu et al. designed an electrochemical sensor based on a dual amplification strategy of polymethylene blue nanoparticles. They tested its efficiency in human serum and food and quantitatively detected from 10 to 10^8^ CFU/mL, with an LOD = 1 CFU/mL [114].

The bacteria responsible for the infection listeriosis (*L. monocytogenes*) is one of the most virulent foodborne pathogens and can produce death. Some current detection strategies include a nanoparticle cluster modified with polylysine, capable of detecting *L. monocytogenes* in the linear range of 5.4 × 10^3^–10^8^ CFU/mL, with an LOD of 5.4 × 10^3^ CFU/mL [115]. Furthermore, Zhao et al. used a fluorimetric sensor based on MIPs obtained by pickering emulsion polymerization to determine the presence of bacteria (LOD = 10^3^ CFU mL^−1^) in milk and pork samples [115].

Most cases of salmonellosis are mainly caused by food (raw chicken eggs and goose eggs) infected with *S. typhimurium*. This microorganism can infect a broad range of vertebrate hosts, including cattle, sheep, horses, rodents, swine, and humans. Functionalized polymeric magnetic nanoparticles using external Raman reporter molecules (RRM) were used by Chattopadhyay et al. to detect *S. typhimurium* in spiked food products to prevent salmonellosis. The limits of detection of the bacterium were found to be 100 cells mL^−1^ and 10 cells mL^−1^ using an 4-mercapto benzoic acid (RRM1) and 5,5′-dithiobis(succinimidyl-2-nitrobenzoate) (RRM2)-based immunosensor, respectively [116]. In addition, the detection of different bacteria using the same system was possible, according to Wu et al. [104]. They used an aptasensor based on colorimetric nanoparticles modified with poly(acrylic acid). Using it, they determined *S. aureus*, *Vibrio parahemolyticus* (a bacteria that causes gastrointestinal illness in humans when ingested in undercooked seafood), and *S. typhimurium*, in aqueous solution (LOD of 25, 10, and 15 CFU mL^−1^, respectively).

#### 2.2.9. Other Targets 

The use of sensor polymers in the detection of species, whether in the food field or any other area, is inadvertently related to dangerous or harmful targets for health. However, polymer sensors can also be used to detect targets that represent a benefit for health or enhance some of the properties of the food, such as taste, color, smell, etc. For example, quercetin is the best-known flavonoid polyphenol and is commonly used as a model for this family of organic compounds in most fruits, vegetables, and flowers. This compound has highly beneficial health properties and cardiovascular protection, including anticancer activity, anti-allergy activity, anti-ulcer properties, antiviral and antibacterial activity, and anti-inflammatory effects. Therefore, it has become a target of interest from the point of view of the quality control of specific products. Thus, Wang et al. designed a sulfur–nitrogen co-doped carbon nanoribbon (SNCNR) polymer, which can detect quercetin by forming Meisenheimer-like complexes. The mechanism of interaction of the sensory polymer and the target is by π–π stacking and electrostatic interaction, and a linear response was obtained in the range of 50.0 nM to 200 μM. The obtained LOD was 21.13 nM [117]. Similar articles describe the development of sensory polymers for the detection of polyphenols in wines and honey [118,119], or diastase activity in honey samples [120].

Returning to dangers or harms to the health of target species, it should be said that each food can have its own biomarkers, and therefore this section would be boundless. However, we have focused on the most relevant publications that cannot be included in the previous sections.

Accordingly, pesticides and mycotoxins are two of the most significant targets in the wine industry. Europe is the world’s largest wine producer, and these compounds are increasingly worrying producers and consumers. Pérez-Ortega et al. designed a sensory system based on solid-phase extraction (SPE) polymeric cartridges (reverse-phase sorbents), specifically two commercial brands, Bond Elut^TM^ Plexa SPE cartridges and Oasis HLB^TM^ SPE cartridges. The first contains a polymeric architecture with a non-retentive, hydroxylated, amide-free surface and a non-polar poly(styrene-divinyl benzene). The second includes a highly hydrophilic, water-wettable polymer with a unique hydrophilic–lipophilic balance. With this sensory system, 9 mycotoxins and 60 multiclass pesticides were studied, and LODs below 1 mg L^−1^ for 87% of the studied compounds were obtained. In addition, the study culminates with a proof of concept with 24 red wine samples from different regions of Spain [121].

*N*-nitrosamines are carcinogenic compounds that can be present in food, drugs, air, water, and soil, so their control and detection is also of great importance. Lu et al. have designed metallocalix[4]arene polymers for the gravimetric determination of these compounds. *N*-nitrosodimethylamine was used as a model compound for the study, and two different polymers having calix[4]arene or 4-tert-butylcalix[4]arene tungsten–imido complexes were tested. The sensory system can detect the target in the air using gravimetric detection on a quartz crystal microbalance. The LOD of the system was 5 ppb [122].

Finally, Hu et al. have developed an ingenious method to detect acrylamide, a potentially carcinogenic substance formed in food after high processing temperatures. They first modified quantum dots with *N*-acryloxysuccinimide. Afterward, the polymerization of acrylamide occurs, and this novel fluorescence detection method relies on the distance increment between quantum dots induced by the polymerization. The system has a linear range of 3.5 × 10^−5^ to 3.5 g L^−1^ and an LOD of 3.5 × 10^−5^ g L^−1^, and the methodology was tested with potato chips [123].

### 2.3. Water and Beverage Treatment. Extraction (Elimination) of Target Species

From a conceptual point of view, there is a big difference between purification/extraction and detection. Consequently, we have separated these two applications into two different sections. When an extraction occurs, the polymers interact with the target, and a high removal capacity is always sought. On the other hand, when detecting a target, a change in some measurable property of the system must be produced in addition to the polymer–target interaction. Therefore, the systems described below simply interact with the target species with the sole objective of removing as much as possible from the medium in which it is found, in this case, water and beverages.

#### 2.3.1. Desalination

The desalination of seawater is usually performed using aromatic PA-based membranes. However, they present some challenges, such as fouling or low permeability. Recently, various approaches have been addressed within this framework to improve the properties of this type of PA-based membranes.

The fabrication of new hybrid organic–inorganic materials through the integration of nanoparticles in polymeric matrices has offered a new strategy for preparing membranes with higher permeability, high selectivity, and better antifouling characteristics. Bano et al. prepared integrated PA nanofiltration (NF) membranes with different graphene oxide (GO) contents. The flux of the new membranes was 12 times higher than in its 100% organic version, and the antifouling properties of the membrane were significantly improved due to an increase in hydrophilicity. These properties were evaluated against bovine serum albumin and humic acid [124]. Similar studies were carried out in which a thin-film composite nanofiltration membrane was prepared with a wrinkled polyamide layer, prepared by interfacial polymerization on a support composed of carbon nanotubes/polyether sulfone [125]. The membrane presented a permeability of up to 53.5 L m^−2^ h^−1^ bar^−1^ with a rejection greater than 95% for Na_2_SO_4_.

Another alternative to traditional PA membranes are the Turing-type membranes. Turing structures appear when imbalances in diffusion rates make a stable steady-state system sensitive to minor heterogeneous perturbations. Tan et al. prepared Turing-type PA membranes by interfacial polymerization and obtained membranes with à la carte shapes, such as bubbled or tube structures. Water transport through membranes and their performance is excellent and promising. Some authors have reported excellent results using polymers different from PAs for water desalination, such as poly(2-(diethylamino)ethyl methacrylate) and poly(2-(dimethylamino) ethyl methacrylate). The microgels showed a water flow of 56 L·m^−2^·h^−1^ and a water recovery of 50% [126]. In another example, Lu et al. developed a methodology for adjusting the permselectivity of cellulose triacetate membranes by a swelling and deswelling procedure induced by plasticizers added to the formulation of the membranes. Membranes demonstrated a decrease in water and salt permeability due to reduced crystallite size in the crystalline regions and chain mobility in the amorphous regions. Since the salt permeability was higher than water, the result was a higher permselectivity and an improved desalination performance. The development allows the design of polymeric membranes with properties of interest in the field of desalination [127].

Zhang et al. have found an ingenious way of applying polymer science and technology in the desalination of seawater. In this case, the technique was capacitive deionization, an emerging eco-friendly technology with high energy efficiency and low operating cost [128,129]. The study focuses on electrode cleanliness, precisely electrode antifouling properties, which are enhanced when a poly(sulfobetaine methacrylate) brush coating is prepared by surface-initiated atomic transfer radical polymerization. These brushes clean the electrode and prevent dirt from embedding in them for at least 100 desalination/regeneration cycles [130].

**Figure 9:**
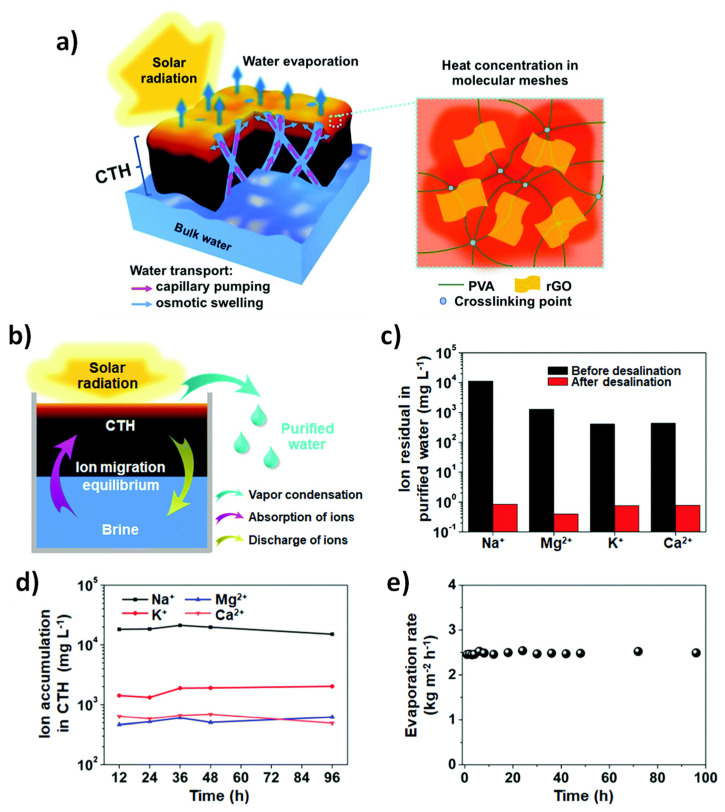
Hybrid hydrogel capillarity aided water transport for solar vapor generation: (**a**) powered by solar energy, intense water evaporation (blue arrows) occurs. Floating hydrogel transports water from the bulk to the evaporation surface, reducing water loss over evaporation. Reduced graphene oxide (rGO) absorbers harvest, transfer, and confine solar energy to the molecular mesh on the surface; (**b**) schematic representation of the solar desalination system; (**c**) concentration of the main ions in seawater before and after desalination; (**d**) concentration of the ions collected in the system over time; and (**e**) duration test of the system for 96 h based on constant solar desalination. Adapted with permission from [131]. Copyright © RSC Publishing 2008.

In addition to nanofiltration membranes, there are other methods for water desalination. Solar desalination is a sustainable method with a great future, but it requires high-rate solar evaporation, which is generally difficult to achieve, and the process is inefficient. Zhou et al. have designed a polyhydrogel that works as a solar evaporator able to generate steam at ~2.5 kg m^−2^ h^−1^ under solar irradiation of 1 kW m^−2^. The hydrogel is based on EVOH and reduced graphene oxide (Figure 9). The former diminishes the enthalpy of water evaporation, and the latter is a solar absorber that improves efficiency. In addition, this hybrid material contains internal capillary channels that allow continuous steam generation and excellent antifouling properties, resulting in an efficiency of about 95% [131].

#### 2.3.2. Toxic Metals

**Figure 10:**
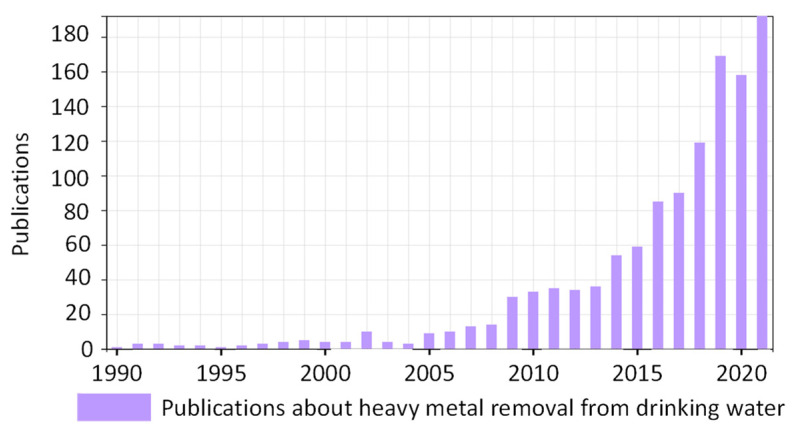
Evolution of the number of publications about heavy metal removal from drinking water from 1990 to 2021. Source: Web of Science.

Some heavy metals (such as arsenic, lead, mercury, and cadmium) are included in the World Health Organization’s list of Chemicals of Major Public Health Concern [132], and they can be found in tap water. Therefore, their extensive use causes environmental contamination, and, in addition, they are cumulative toxicants that affect multiple body systems, including gastrointestinal, cardiovascular, renal, neurologic, and hematologic systems. Consequently, the interest in eliminating heavy metals from drinking water produces countless publications yearly. This number was multiplied by six in the last decade, as shown in Figure 10, with reported methods based on adsorption; membrane; and chemical, electric, and catalytic treatments.

Each metal and relevant publication has specific properties and particularities, making it very difficult to deal with this section from a general point of view. Therefore, we have decided to summarize this large amount of information in Table 3, which indicates the type of metal (target), the polymer type, and some observations regarding the amount of metal that can be removed.

#### 2.3.3. Denitrification

Nitrates can end up in drinking water mainly because of their extensive use in fertilizers, manure, and liquid waste discharge from septic tanks carried by rain or irrigation. The denitrification process (removal of nitrates from water) is complex. Despite the many techniques that can be used, three of them are the most relevant from a polymer science point of view, namely, the use of bioreactors, adsorptive membranes, and ion-exchange materials.

Chu et al. designed a fixed bed bioreactor loaded with a PCL biopolymer, which acts as a carbon source and biofilm carrier. During the study, the average NO_3_^−^ in the effluent was less than 3.7 mg N L^−1^, and more than 95% of total nitrogen was withdrawn at a hydraulic retention time of 3–6 h [148]. The same authors carried out similar studies by using three kinds of biopolymers blends (poly-3-hydroxybutyrate-co-hyroxyvelate (PHBV), PHBV/starch, and PHBV/bamboo powder) as carbon source and biofilm carriers, obtaining removal efficiencies of about 90% [149].

**Figure 11:**
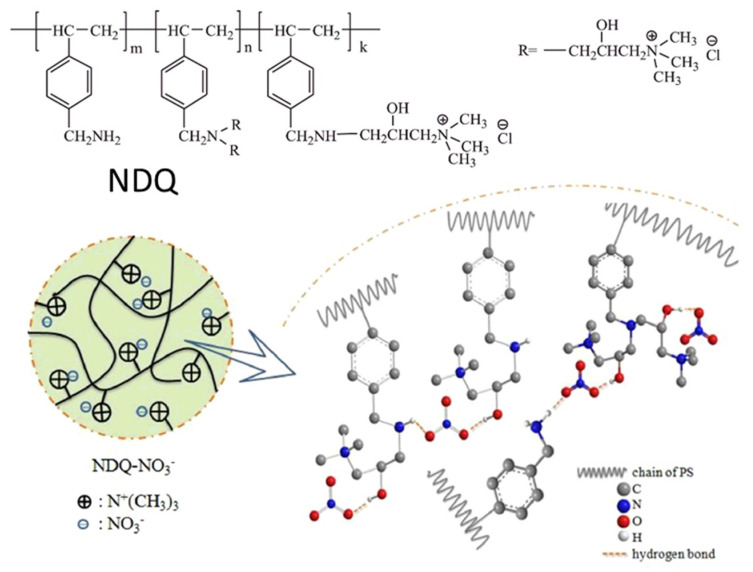
PS modified with amino and quaternary ammonium copolymer (NDQ) and nitrate binding mechanism through hydrogen bonding. Adapted with permission from [152]. Copyright © Elsevier 2016.

Regarding ion-exchange materials, Nabid et al. found a new use for conducting polymers as stable nanocomposites for nitrate ion exchange materials [150]. The nanocomposites were made of multi-walled carbon nanotubes with different polymers (poly(1,8-diaminonaphthalene), polypyrrole, PAni, and poly(2-vinylpyridine)). This way, removal efficiencies around 1.20 g L^−1^ were reached at pH = 6.5 and ambient temperature. In other relevant publications, different polymers were used with the same aim as cellulose nanocrystals (CNC)-grafted copolymers of 2-(dimethylamino)ethyl methacrylate and coumarin monomers [151], PS modified with amino and quaternary ammonium groups (Figure 11) [152], chitosan/PEG and chitosan/PVA polymer composites [153], and polyacrylonitrile–alumina nanoparticle mixed-matrix hollow-fiber membranes [154].

#### 2.3.4. Fluoride Elimination

Fluoride is present in natural rocks (sellaite, cryolite, fluorapatite, etc.), but the high levels found in water are directly caused by human actions, such as the excessive use of phosphate fertilizers. The International Organization for Standardization proposes acceptable fluoride values in drinking water from 0.5 to 1.0 mg L^−1^. However, many Asian countries show 35 times higher concentration values in drinking water, which is alarming. Moreover, both excess and deficiency of fluoride can lead to health problems, such as dental caries or skeletal fluorosis, respectively [155]. For this reason, this subsection aims to point out this new target detected in the present review related to water. It is an emerging society-concerning target whose removal is already beginning to be studied.

Consequently, diverse technologies have been developed to reduce the fluoride amounts in water, such as coagulation, adsorption, precipitation, and membrane separations. However, pressure-driven polymeric membranes made of PAs, polyvinylidene fluoride, polyurethane, polyacrylonitrile, and polysulfone are the most relevant technologies from a polymer-science viewpoint [155].

### 2.4. Polymers for the Separation of Targets

The main objective of these polymers is to interact with specific target species in such a way that they are extracted from the environment in which they were found. Unlike polymers defined in Section 2.3, this interaction between the target species and the polymer must be reversible, and both the target and the polymer must be recoverable to quantify the concentration in external equipment. However, in this section, we will focus only on the extraction procedure, which is this review’s aim, and not on the quantification techniques used.

MIPs are probably the most relevant family for solid-phase extraction (SPE), solid-phase microextraction, dispersive SPE, stir bar sorptive extraction, magnetic separation technology, etc. Applied to food technology, there are some relevant publications in the last decade, as published by Jiang et al. They reported the separation and determination of carbohydrates in food samples (milk, honey, juice, candied jujube, beer, and chitosan oligosaccharide capsule). Under the most favorable conditions, 32 carbohydrates, including mono, di, and oligosaccharides and sugar alcohols, were separated in less than 12 min. The technique carried out was capillary electrophoresis, but the key was the use of a cationic polymer of hexadimethrine bromide as an electroosmotic flow reverser [156].

Another MIP-based publication was reported by Wang et al. for the separation of glibenclamide in health foods, a drug for treating type 2 diabetes. The MIPs were prepared using dendritic-grafting magnetic nanoparticles, with methacrylic acid (functional monomer) and with a cross-linker (glycol dimethacrylate), rendering the recovery of glibenclamide in spiked health foods of 81.46–93.53%, with an RSD < 4.07% [157]. Similar works have been published for the separation of zanthoxylum alkylamides from prickly ash powder [158]; sunset yellow (E110) from fruit juice, fruit juice powder, and pharmaceutical samples [159]; dichlorodiphenyltrichloroethane [160] and cadmium [137] from various food samples; mercury(II) ions from fish samples [77]; lead from aqueous samples [161]; or citrinin from maize [162].

However, in recent years, promising competitors have emerged for MIPs, such as open-tubular capillary electrochromatography (OT-CEC) using grafted polymers. Specifically, two publications stand out in the literature, in which Aydoğan et al. describe the preparation of open tubular columns by the in-situ grafting polymerization of 3-chloro-2-hydroxypropyl methacrylate (HPMA-Cl) for the separation of malic acid amino and acids enantiomers [163,164]. In the first case, the polymerization of HPMA-Cl is followed by a modification with l-histidine and in the second case with β-cyclodextrin (β-CD).

## 3. Classified by Type of Polymer (Polymers Widely Used in Food Packaging)

This section aims to summarize the polymers primarily used on a large scale within the food industry, paying particular attention to food packaging.

### 3.1. Polyethylene Terephthalate (PET)

PET is a polyester formed on the polycondensation of terephthalic acid and ethylene glycol, which are derived from oil feedstock. It can be prepared with high crystallinity (up to 44% in drawn fiber [165]). Still, it is normal to find lower crystallinities in food packaging (around 21% in water bottles [166]), since higher crystallinities would unbalance the ratio between mechanical properties (impact properties and thermal stability) and transparency by decreasing the latter.

Although large companies commercialized it, the discovery of this polymer is attributed to chemists Whinfield and Dickson, employees of the Calico Printers’ Association of Manchester.

In general, all the polymers used in food packaging require very high purities, and given the complication of purifying a polymer, the reagents are usually purified by high vacuum distillation (ethylene glycol) and recrystallization (terephthalic acid). As a catalyst, antimony is generally used, although it is common to use other metals such as titanium, germanium, and aluminum; in any case, the amounts are always extremely low.

PET is mainly used in food packaging in three formats: bottles, semi-rigid sheets for trays, and thin-oriented films for sandwich wrappers. The use defines the requirements, which in turn establishes the production method, as depicted in Table 4 [167]. PET is extensively used for beverage packaging due to its higher resistance than other types of plastics to CO_2_ permeation losses. In addition, it is plastic with low diffusivity (that is, it does not allow the critical diffusion of organic compounds into the plastic) [168]. That is why one or even three layers of PET are used for containers oriented to beer or carbonated drinks.

**Figure 12:**
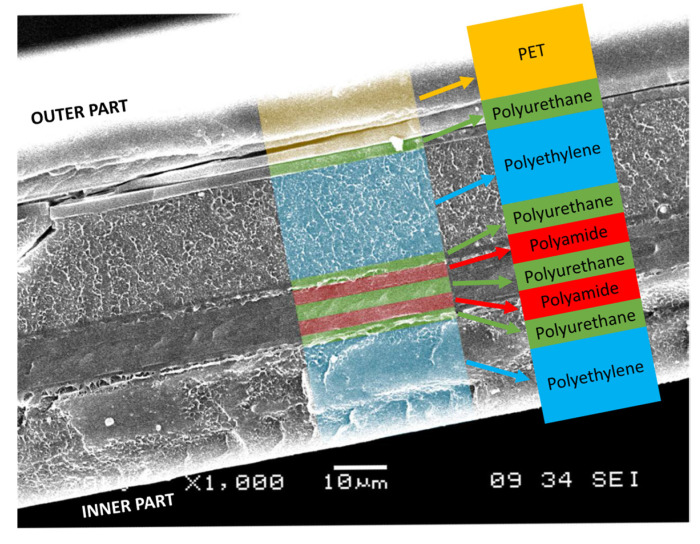
Multilayer film for food packaging applications.

In multilayer packaging, which is usually used in processed meat products (sausages, mortadella, etc.) or pre-cooked products (pizzas, cooked cold cuts, etc.), it is generally placed in the outermost layer. In addition to its barrier properties, this is also done because it is an easy-to-paint polymer wherein brands can place their logos. Figure 12 shows a cross-section of an actual nine-layer film for food packaging.

PET is also a very suitable polymer for recycling. In fact, it is recycled the most, in tons per year, worldwide, which also gives an approximate view of its manufacturing volume.

Regarding food contact regulations, European Union established that “any material or article intended to come into contact directly or indirectly with food must be sufficiently inert to preclude substances from being transferred to food in quantities large enough to endanger human health or to bring about an unacceptable change in the composition of the food or a deterioration in its organoleptic properties” (European Commission No 2023/2006). Specifically for plastic materials, Regulation (EU) No 10/2011 (and subsequent amendments) states that: (1) there are lists of authorized substances, including starting reactive monomers and additives; (2) there is an overall migration limit (OML) for the sum of all substances in the food product; (3) there are specific limits (SML) for certain substances, depending on their dangerousness (Table 5) [167].

### 3.2. Polyethylene (PE)

PE is a thermoplastic polymer and probably the best-known and most-used polyolefin globally. It was accidentally discovered in 1933 by scientists of the ICI laboratories in the UK in the form of what is now known as LDPE [169]. Today, many more types of PE are produced depending on their density, such as HDPE, medium-density polyethylene (MDPE), very-low-density polyethylene (VLDPE), LLDPE, ultra-low-density polyethylene (ULDPE), high-molecular-weight polyethylene (UHMWPE), and cross-linked polyethylene (XPE). The differences in the density of PE are a direct consequence of its degree of crystallinity. A common LDPE can have a density of 0.915–0.940 g/cm^3^, with crystallinities of 45–55% and molecular weights of 10–50 kDa. However, HDPE can reach molecular weights up to 250 kDa, with degrees of crystallinity of 70–90% and densities of 0.940–0.970 g/cm^3^. The melting point just above 100 °C prevents it from being used in food containers that will suffer sudden heating or high temperatures.

All polyolefins are susceptible to oxidative degradation processes that result in chain branching. Therefore, antioxidants such as phenol and phosphite derivatives are usually added as additives at low concentrations (0.01 to 0.5 wt%) [170]. Depending on the final product, the additives are selected à la carte for each format. For example, when manufacturing films, slip agents such as fatty acid amides (oleamide and erucamide) are often added, diffusing to the film’s surface after manufacturing. Titanium dioxide and calcium carbonate are very recurrent additives since they make the final product cheaper and provide a white color characteristic of many bags and bottles. Other additives of interest are polyethylene glycol esters, glycerol monostearate, and ethoxylated secondary amines, which are added to PE formulations as antistatic agents, or sodium alkane sulphonates, added as lubricants.

PE is mainly used in food packaging in two formats, films and bottles/containers [169], as depicted in Table 6.

In multilayer packaging, it is usually placed in the innermost layer, in contact with the food (Figure 12). However, PE is not an easy-to-paint plastic, and therefore, when there is no other choice, different techniques are used (corona discharge, flame, and ozone) to increase the surface energy and allow painting or printing.

Regarding regulatory aspects (European Commission No 2023/2006 and No 10/2011), Table 7 collects the most relevant information regarding the migration of additives from PE.

### 3.3. Polyvinyl Chloride (PVC)

PVC is a long-chain polymer produced by free radical polymerization in an autoclave, obtaining theoretical molecular weights of around 30 to 95 kDa. The largest proportion of PVC in contact with food is the unplasticized PVC (PVC-U), as trays and containers produced from sheets extruded by thermoforming process (around 50%) and as bottles (35%). On the other hand, plasticized PVC (PVC-P) plays a relevant role in applications such as cling film (11%) and repeated-use closures and hose applications [171].

PVC is a self-extinguishing polymer since it releases HCl during combustion, displacing oxygen from the combustion process and extinguishing the flame. However, PVC is thermally quite unstable, so organotin-based thermal stabilizers are one of the main additives. Non-plasticized PVCs usually require additives such as the terpolymer of methacrylate–butadiene–styrene (MBS) to improve their impact properties. Other additives do not affect the final product properties but greatly help the processing of the polymer, such as acrylic polymers and methacrylic–styrene copolymers. As the most common plasticizers in the production of PVC, we find phthalates, adipates, and trimellitates. As we focus on the food industry, epoxidized soybean oil and adipates stand out. Finally, the most-used lubricants are glycerol monooleate, PE wax, stearic acid, and high-molecular-weight mineral oils.

PVC was the first option for the packaging of beverages after what was established by the Association of Plastics Manufacturers of Europe in 1990, mainly because of its low cost and the chance of manufacturing very resistant and low-weight containers. However, it was soon overshadowed by PET, which offers lower CO_2_ permeability. Today, PVC is mainly used in food packaging in the applications shown in Table 8.

Regarding regulatory aspects (European Commission No 2023/2006 and No 10/2011), Table 9 collects the most relevant information regarding the migration of additives from PVC.

### 3.4. Polypropylene (PP)

After PE, PP is the most widely used polyolefin in food packaging. The main difference is that PE can be obtained in three different forms from the point of view of the chemical structure and, more specifically, from the point of view of the side-chain methyl group: isotactic, syndiotactic, and atactic. The PP found in everyday applications is isotactic. Syndiotactic PP is less crystalline and is only used as elastomers. On the other hand, atactics are highly amorphous, resulting in sticky polymers used as hot melt adhesives [172].

Generally, the additives commonly included in PP formulations are the same as those for PE, i.e., phenolic, thioether, and phosphite-based antioxidants; oleamide and erucamide as slip agents; calcium carbonate and titanium dioxide as fillers and white dyes, etc. The only difference is related to the stereoregularity of the PP, which in the case of the PE, does not exist. This property is improved when nucleating agents are added to favor crystallinity. The most used are benzoates (sodium, potassium, aluminum) and sorbitols, such as bis-benzylidene sorbitol, which are included in concentrations of up to 0.5%.

Propylene can be used in different applications in the food industry: (1) pots/containers for yogurt, soups, cottage cheese, margarine, chilled salads, desserts, sauces, or pot noodles; (2) cast-film bags for bread; (3) bags/wraps made of OPP for snacks, biscuits, confectionery, etc.; (4) OPP film overwraps for cooked meats, vegetables, or tea; (5) multilayer lidding films for packaged meat and fish; (6) film pouches for sauces and liquids; (7) bottles and caps for sauces and soft drinks, respectively; and (8) paperboard/PP laminates for dairy products and ready meals for microwave heating.

### 3.5. Polystyrene (PS)

Both PS and styrene–butadiene copolymers have been major players in food packaging for decades, and their foamed trays are characteristic of meat, fish, fruit, etc. In addition, styrofoam will not rot or mildew, and its moisture resistance makes it ideal for keeping such products fresh [173].

The lateral aromatic rings of its structure represent clear differences from other members of the polyolefin family, such as PE and PP. In addition, PS has a glass transition temperature of about 100 °C and an amorphous nature, which makes it a perfect material for injection molding. However, PS has weak barrier properties to water vapor and gases, such as oxygen and carbon dioxide. Table 10 summarizes the main additives used in the manufacturing of PS.

Regarding its applications in the food industry, there are two significant limitations, one of them related to the temperature of use (it cannot be used to cook food in the oven) and the other to the storage of foods rich in fat, due to the tendency to stress crack when in contact with such foods. As has been said before, the flagship application of PS is as foamed PS, or XPS, trays. Some PS trays, cups, and containers have surface layers of crystalline PS that provide a “barrier” layer between the plastic and the food. The apparent density of these trays ranges between 0.05 and 0.19 g/cm^3^, and thicknesses between 0.3 and 6.4 mm are used. There are also biaxially oriented PS films, often used as thin transparent windows in paperboards or as “breathable” films for wrapping vegetables. Increasing the thickness, sheets are obtained for manufacturing cups and tubs for desserts and preserves through a thermoforming process.

High-impact polystyrenes (HIPS) are produced from a styrene–butadiene copolymer and crystalline PS blends. It is precisely the balance between these two polymers that gives manufacturers the option to tune the forms of packaging processes: injection-molding or thermoforming. For example, these PS derivatives are used as yogurt containers or coffee cups for vending machines.

### 3.6. Polymers in Printing Inks for Food Packaging

There are many compositions for printing inks. Still, classical polymers have a relevant role in four of them: gravure and flexographic printing (solvent and water-based systems), UV-curing inks, and digital printing (non-impact printing).

Gravure and flexographic printing could be carried out with solvent-based systems, in which nitrocellulose is the primarily used binder. However, maleic resin, polyvinyl butyral, PA, and polyurethane are strong alternatives to nitrocellulose. On the other hand, water-based systems use styrene–acrylic co-polymers and acrylic co-polymers, as binders (copolymers of acrylic acid, usually).

Related to UV-curing inks and lacquers, epoxy acrylates, polyester acrylates, polyether acrylates, and urethane acrylates are used as oligomers and PE/PTFE waxes or silicone oils as additives. The toner is based on pigments mixed with styrene, acrylate, or polyester, which act as a carrier for iron oxide particles, which are transferred electromagnetically to the imaging drum [174].

With the rise of additive manufacturing, production and research on polymers in inks for 3D printing, especially polysaccharides, have also been boosted. The latest advances in inks reveal a growing interest in these polysaccharide-based inks, as extra functionalities, such as antimicrobial properties, can be included in the inks [175]. Xanthan, gellan, dextran hyaluronic acid alginate, or bacterial cellulose are only a few examples of polysaccharides used in 3D printing to provide different functionalities.

### 3.7. Polymeric Adhesives for Food Packaging

Polyurethanes (PUR) are relevant polymeric adhesives in food packaging and indispensable in the production of flexible packaging. They have high flexibility and bonding strength. Food containers with polyurethane adhesives are generally intended for use at low temperatures (e.g., coffee powder bags and chips) and at room temperature. However, in some cases, they have also been designed to withstand high temperatures. When PURs are used as adhesives in food packaging is essential to control the correct curing, respecting time, temperature, and humidity. Failure to fully cure carries the risk of migration of primary aromatic amines [176,177].

Natural polymers are increasingly present, and their use as adhesives in food packaging is booming, although their curing is usually slow. Water-based adhesives containing starch, dextrin, and/or casein are often used for label adhesion. Casein adhesives are ideal for labeling cold and wet bottles and returnable glass bottles, since they are in aqueous caustic soda. These adhesives are biocompatible and are also often used for bonding toilet paper or wrapping cigarette leaves [176].

Another group of adhesives widely used in food packaging is those based on PVAc. Their most important applications are the sealing of paper containers (sugar bags), cardboard and corrugated cardboard (CB) (fruits, vegetables), folding boxes (side seam), bags (bread), etc. We can differentiate two large groups within this, those that use PVAc homopolymers and those that use copolymers. The former requires plasticizers and is mainly used in secondary packaging applications. The latter is used for primary packaging and are preferable when bonding two substrates of different natures [176,178,179].

Hot melts are used for paperboard and case closing, tray erection, the attachment of straws, or bonding of plastic caps onto bricks. They are very useful in high-speed machines, where hot tack and excellent sealing capabilities are required. These adhesives work very well with substrates such as paper, film, cardboard, aluminum, or combinations thereof [176,180].

Coldseals are typically used for packaging temperature-sensitive foods (ice cream, chocolate, etc.) They can be used in temperatures between 15 and 25 °C. On the other hand, heatseals are used on products with disposable lids, such as yogurts. These adhesives must ensure the safe transportation of the food but must also be easy to remove by applying reasonable force. Table 11 summarizes all adhesives used in food packaging [176].

## 4. Classified by Environmental Hazard

Every year, 380 million tons of plastics are generated throughout the world, oriented to packaging (39.6%), construction (20.4%), automotive (9.6%), electronics (6.2%), home (4.1%), agriculture (3.4%), and others (16.7%). Considering Europe as an example, 55 million tons are generated annually, of which only 29 are collected: 23% end up in landfills; 42% is burned, and 34.6% is recycled [16]. Recycling and reuse continue to be the best solutions for the problem of plastic pollution since they allow them never to become waste, i.e., they value waste.

Regrettably, plastic pollution is a highly topical issue that concerns both citizens and governments. The arrival of plastic in the food chain has been related to the reduction of migration and propagation of human mesenchymal stem cells from bone marrow and endothelial progenitor cells [181]. Moreover, each plastic has a chemical structure directly related to its recycling and degradation or biodegradation. For example, regarding PET, Dhaka et al. studied its harmful effects as macroplastic, mesoplastic, microplastic, and nanoplastic in different environments (groundwater, drinking water, soils, and sediments). As the most relevant conclusions of the work, it was extracted that the ingestion of PET microplastics by vertebrates and invertebrates is the main reason for their lethal inner injuries.

There is great interest in recycling all types of plastic, but PET is particularly relevant for the food industry since it is easy to recycle and authorized for food contact uses after recycling [181,182]. Table 12 summarizes the ease with which the most widely used plastics worldwide and, therefore those of greatest concern from an environmental point of view, can be recycled.

**Figure 13:**
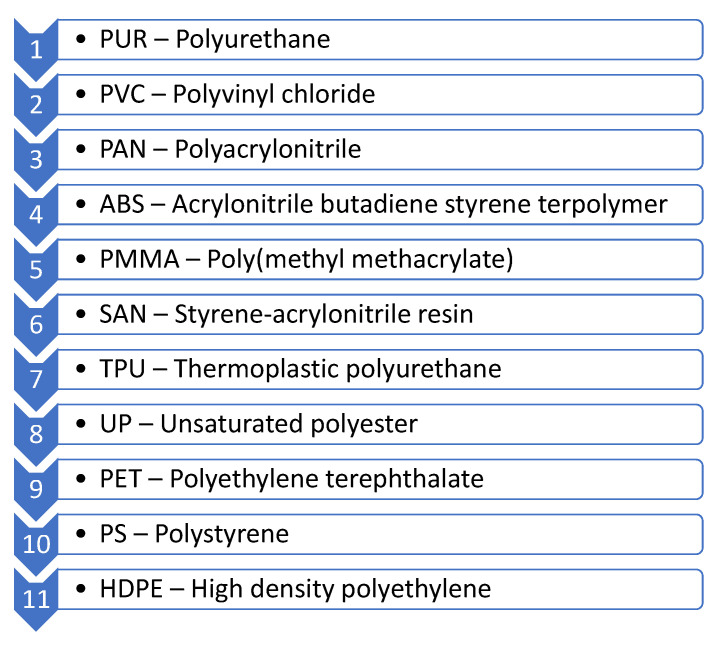
Hazard scale of plastic microparticles.

Many publications treat microplastics as a single set, a single contaminant. However, the problem should be studied for each type of plastic, as done by different authors, who systematically explored the potential implications of the predominant polymers in the environment and their associated additives [182,184]. Yuan et al. developed a semi-quantitative risk assessment model, which allows classifying the health hazard of the different plastic microparticles, focusing on those that enter the food chain through marine exposure [184]. The categorization was done using a method based on three probability factors and two impact factors. Although many polymers were studied, the study focused on those of the most significant concern and those shown to give rise to the most dangerous microplastics. Figure 13 shows the hazard scale of plastic microparticles according to the categorization done by Yuan et al. The conclusion was drawn from the study that the plastic microparticles of these polymers are the most significant concerning the potential risk to human health from food chain exposure pathways influenced by marine waters.

Lithner et al. have carried out similar classification works, even from a broader viewpoint, analyzing not only many polymers but also the dangerousness of their monomers [185]. Furthermore, this study does not focus only on microparticles but on materials in general. This classification matches the previous one in several of the most dangerous polymers. The authors identified PUR, polyacrylonitriles, PVC, epoxy resins, and styrene copolymers (acrylonitrile–butadiene–styrene terpolymer, ABS; styrene–acrylonitrile copolymer, SAN; and HIPS) with the highest level of danger. Furthermore, considering the polymers in the second highest level of danger (phenol–formaldehyde resins, unsaturated polyesters, polycarbonate, polymethyl methacrylate, and urea–formaldehyde resins, etc.), a total of 31 of the 55 polymers studied are highly dangerous. In addition, special mention is made of PVC, since it is the third-most-used plastic and since its monomer is carcinogenic.

## 5. Conclusions/Prospects

For years, polymers have contributed to food safety and security by protecting against manipulation, damage, and contaminants and the extension of the food shelf-life, mainly as innovative and intelligent packaging prepared with multilayer materials with à la carte barrier properties but also as edible polymers with similar characteristics. Lately, advanced polymers with smart abilities are opening new opportunities for providing additional security and shelf-life extension of foodstuff related to advanced food packaging, the detection and quantification of target chemical species, and food treatment. On the other hand, the success of polymers in this matter brings the continuous volume increment of generated waste, which means challenges in recovery and recycling, that must be tackled by researchers and industry in the forthcoming years and in parallel opportunities from research and industrial viewpoints.

## Figures and Tables

**Table 1:** Classification of the most relevant publications of the last ten years about active packaging through chemical species delivery, identifying the delivered drug, the host polymer, and some comments about the obtained results.

Delivered Drug	Host Polymer	Comments	Ref.
Tocopherol	PP films modified with different chain extenders	In films with antioxidant activity, tocopherol is released as temperature and storage time increase. Chain extender migration tests fall within the European legislation’s values.	[22]
Silver and titanium dioxide nanoparticles	PLA	Active agents, including Ag nanoparticles, TiO_2_ nanoparticles, and their combination were incorporated in various films to perform a proof of concept to test the life cycle. The results demonstrated that a film with both nanoparticles have fewer environmental effects.	[23]
Nisin	EVOH-based nanofibers	Nisin release from the nanofiber was well controlled, following the Fickian diffusion model and showing improved antimicrobial effect against *Staphylococcus aureus*.	[24]
Water soluble protein	PS	Water-soluble protein was encapsulated for the first time using hydrophobic PS. The authors used an electrospinning procedure, taking advantage of L-limonene for the controlled release of a model protein (BSA).	[25]
Thymol	Blend of chitosan/quinoa protein	Antimicrobial activity was tested against *Listeria innocua*, *S. aureus*, *Salmonella typhimurium*, *Enterobacter aerogenes*, *Pseudomonas aeruginosa*, and *Escherichia coli.*	[26]
Essential oils (carvacrol, thymol, and eugenol)	LLDPE-based active clay nanocomposite	Antimicrobial activity against *E. coli* was investigated in fresh beef and fermented Turkish-type sausages. The color of fresh meat was maintained for up to 4 days.	[27]
Essential oils from clove bud and oregano	Methylcellulose	Both essential oils reduced the stiffness of methylcellulose films and reduced yeast and mold counts in sliced bread for 15 days.	[28]
Olive oil and ginger oil	Composite (bacterial cellulose, carboxymethylcellulose, and glycerol)	The composite film’s antimicrobial activity was studied for nine weeks (at different temperatures) against *Staphylococcus aureus*, *Pseudomonas aeruginosa*, *Escherichia coli*, *Candida albicans*, and *Trichosporon* sp.	[29]
Carvacrol and thymol	PP films	Compared to carvacrol, thymol showed higher antimicrobial activity, inhibiting bacterial growth in food.	[30]
Anthocyanins and limonene	Starch/PVA	The system showed simultaneous color change and antimicrobial activity. The system revealed outstanding antimicrobial activity for *Bacillus subtilis*, *Aspergillus niger*, and *S. aureus* tested in pasteurized milk.	[31]
Essential oils: clove leaf oil, sweet basil oil, and cinnamon bark oil.	LDPE/EVA blended films	The system was tested with sliced tomatoes and showed good inhibition % for *E. coli* and *S. aureus.*	[32]
Ethanolic extracts of cinnamon, guarana, rosemary, and boldo-do-chile	Gelatin and chitosan	High growth inhibition % for *E. coli* and *S. aureus*	[33]
*Origanum vulgare* L. essential oil		The films revealed a unique antimicrobial effect for common food pathogens: *S. aureus*, *Listeria monocytogenes*, *Salmonella enteritidis*, and *E. coli.*	[34]
Lignin	PVA and chitosan	The system showed Gram-negative bacterial growth inhibition against *Erwinia carotovora* subsp. *carotovora* and *Xanthomonas arboricola* pv. Pruni.	[35]
Silver nanoparticles and organoclay (Cloisite 30B)	Gelatin	The nanocomposites demonstrated both Gram-positive and -negative food-borne pathogen inhibition.	[36]

**Table 3:** Classification of the most relevant works of the last ten years about heavy metal removal from drinking water, identifying the target heavy metal, and the type of polymer.

Target Ions	Type of Sensory Polymer	Comments	Ref.
**Lead**	Poly-melamine-formaldehyde polymer	The polymer has good porosity and thus a high surface area and density of functional groups (amine and triazine). It can rapidly diminish lead ions in water to trace levels (ppt). Other ions commonly found in drinking water do not present interference, such as Na^+^, K^+^, and Ca^2+^. The system was successfully tested with water in dynamic flow. The process is reversible, and the material can be reused.	[133]
**Arsenic**	PAni/Fe0 composite	The maximum adsorption capabilities at pH 7.0 for As(III) and As(V) were 232.5 and 227.3 mg/g, respectively. HCO_3_^−^, SiO_3_^2−^, and SO_4_^2−^ ions did not interfere with the removal, but NO_3_^−^ and PO_4_^3−^ did.	[134]
	Poly(1-vinyl imidazole)-based IIP	Compared to a non-imprinted polymer, the relative selectivity coefficient of MIP for As^3+^/Cd^2+^, As^3+^/Zn^2+^, and As^3+^/Ni^2+^ were respectively 45.93, 131.01, and 262.63 times greater.	[135]
**Copper**	2-thiozylmethacrylamide-based IIP	Quantitative retention was achieved between pH 5.0 and 6.0.	[136]
**Cadmium**	IIPs containing magnetic nanoparticles	Tested in the extraction of cadmium ions from food samples (shrimp, fish, crab, persimmon, apple, tomato, mushroom, and potato)	[137]
**Cadmium and lead**	Poly(2-(diethylamino) ethyl metacrilate) containing 8-hydroxyquinoline motifs	Quantitative retention was achieved at pH 8.5.	[78]
**Mercury**	Copolymer of *N*-vinylpyrrolidone, methylmethacrylate, and a monomer containing dithizone motifs	86% removal	[81]
	Polyvinylidene fluoride membrane with blended MoS_2_ nanosheets	The most favorable pH values for mercury ion removal were 4.5–6.0. Maximum adsorption capacity = 578 mg g^−1^	[138]
	Fluorescent supramolecular polymer; thymine-modified [2]biphenyl-extended version of pillarene	The pillarene serves as host by an easy supramolecular assembly aided by an AIEgen-bridged quaternary ammonium guest.	[139]
	Amorphous porous aromatic framework	Mercury uptake capability of over 1000 mg g^−1^, and the system can efficiently diminish the mercury(II) content from 10 ppm to 0.4 ppb. Removal efficiency = >99.9%.	[140]
	Water-stable metal–organic framework/polymer based on Fe-1,3,5-benzenetricarboxylate and polydopamine	The material binds up to 1634 mg of Hg(II) and 394 mg of Pb^2+^ per gram of composite. Removal % = 99.8% from a 1 ppm solution, with no interference from Na^+^ ions; it is resistant to fouling when tested with humic acid and is fully regenerable over many cycles.	[141]
	Urea–formaldehyde polymer containing polymer bimetal complexes (nickel ferrite bearing nitrogen-doped mesoporous carbon)	NiFe_2_O_4_-NC had a high Brunauer–Emmett–Teller surface area (147.4 m^2^ g^−1^), and the particles were in the range of 8 to 10 nm. The maximum adsorption capacity was 476.2 mg g^−1^ at 25 °C.	[142]
**Lead** **Cadmium** **Niquel**	Modified Fe_3_O_4_ nanoparticles modified with carboxymethyl-β-cyclodextrin polymer	In non-competitive adsorption mode at 25 °C, the maximum Pb^2+^, Cd^2+^, and Ni^2+^ uptakes were 64.5, 27.7, and 13.2 mg g^−1^, respectively, at 25 °C.	[143]
**Zinc** **Cadmium** **Nickel** **Mercury** **Cobalt** **Copper**	Polymer beads containing DMPS–Si-pyronine-based fluorescent probe (DMPS = 2,3-dimercapto-1-propanesulfonic sodium)	The system is valid for determining, detoxifying, and eliminating heavy metal ions. The percent of detection, removal, and detoxification are 98.10%, 97.59%, and 65.55%, respectively. The system could be recycled 10 times.	[144]
**Chromium** **Mercury** **Copper** **Cadmium**	Polymer film based on electrospun polyacrylonitrile containing a zeolitic imidazolate framework-8	The capability for heavy metal removal improved up to three times compared to pure polyacrylonitrile films. Removal efficiency of 99.5%	[145]
**Copper** **Iron** **Manganese** **Zinc**	Fly ash-based geopolymer	The ashes contain mainly alumino-silicate oxide from coal combustion. Basically, it is an inorganic polymer with Si-O-Al polymeric bonds, with an amorphous to semi-crystalline structure.	[146]
**Zinc** **Iron** **Nickel** **Copper**	Coordination polymers (porous materials composed of various metals and suitable organic ligands)	Adsorption efficiency up to 99% and adsorption capability up to 348 mg/g	[147]

**Table 4:** Application for PET-based products and manufacturing technology. Adapted from Table 1 in reference [167].

Manufacturing Technology	Format	Applications
Injection, stretch blow, molding	Bottles Wide-mouth jars and tubs	Bottles manufactured by injection, stretch blow, and molding are often used to bottle water, juices, beer, and carbonated beverages. This type of wide-mouth packaging is very characteristic of products such as jams, preserves, fruits, and dry foods.
Thermoforming	Trays	They are used in precooked products, ideal for heating for a few minutes in a microwave oven, such as precooked pizzas.
Films	Films and metalized foils	These films are used in the manufacturing of packaging for snacks, nuts, sweets, ice cream, etc. On many occasions, it is part of a multilayer.

**Table 5:** Monomers and additives used in PET manufacturing for food packaging. Adapted from Table 2 in reference [167].

Monomers and Additives	Specific Migration Limit (SML)	Function
Terephthalic acid (PTA)	7.5 mg/kg	Monomer
Terephthalic acid, dimethyl ester (DMT)	No SML	
Isophthalic acid (IPA)	5 mg/kg	Additive to enhance processing and performance
Ethylene glycol (EG)	30 mg/kg (Alone or with diethylene glycol or stearic acid esters of ethylene glycol)	Monomer
Diethylene glycol (DEG)		
1,4-Bis(hydroxymethyl)cyclohexane (CHDM)	No SML	Additive to enhance processing and performance
Antimony trioxide	0.04 mg/kg, expressed as antimony	Catalyst

**Table 6:** Application for PE-based products and manufacturing technology [169].

Manufacturing Technology	Product	Applications
Cast and oriented processes	Films	Generally, LDPE is for simple PE films. Boil-in-the-bag foods. It is laminated with PA, but the function of PE is usually heat-sealed and a barrier against polar gases, such as water and CO_2_.
Extrusion coating	Multilayer	Milk Containers. PE is combined with paperboard and aluminum. Packaging for coffee. An aluminum foil layer is incorporated to provide good barrier properties to oxygen. Yogurt and other dairy products. PE/aluminum lidding. Take-away beverages and foods. PE/paperboard/PE laminate containers.
Thermoforming and blow molding	Bottles and other containers	Generally, HDPE for characteristic white bottles.
Miscellaneous	Film bags, heat-sealed overwrapping film, and container liners for bulk transport	Fresh fruit and vegetables Frozen fruit, poultry, vegetables, meat, and fish products Cereals Bread and bakery products

**Table 7:** Monomers and additives used in PE manufacturing for food packaging. Adapted from page 14 in reference [169].

Monomers and Additives	Specific Migration Limit (SML)	Function
Pentaerythritol tetrakis [3-(3,5-di-tert-butyl-4-hydroxphenyl)propionate]	None	Antioxidant
Octadecyl 3- (3,5-di-tert-butyl-4-hydroxyphenyl)propionate	SML = 6 mg/kg	
Phosphorous acid, tri (2,4-di-tert-butylphenyl) ester	None	
Erucamide, oleamide, and stearamide	None	Slip agent
Calcium carbonate, talc, and titanium dioxide	None	Fillers
Glycerol monostearate	None	Anti-static agent
*N,N*-bis(2-hydroxyethyl)alkyl(C8-C18) amine hydrochlorides	SML (T) = 1.2 mg/kg (expressed as *N*,*N*-bis(2- hydroxyethyl)alkyl(C8-C18) amine)	

**Table 8:** Application for PVC-based products [171].

Manufacturing Technology	Product	Applications
1°. Extrusion into thermoformed sheet. 2°. Thermoforming process	Trays and containers	Extended-shelf-life food trays, general-purpose food trays, and collation or straight-on-shelf display trays (PVC-U)
Blow-molding	Bottles	Container for liquids and drinks
Blow-film extrusion	Flexible film	Food preservation in supermarkets and domestic kitchens (PVC-P)
Emulsion polymerization	Coating	Adhesives for closures and can linings. These formulations are known as PVC “plastisols”.
Extrusion	Tubes	Hose and tubing. Transport of soft drinks and beers, etc.

**Table 9:** Monomers and additives used in PVC manufacturing for food packaging. Adapted from Table 2 in reference [171].

Monomers and/or Additives	Specific Migration Limit—SML (mg/kg) According to 2002/72/EC	Function
Organo–tin compounds	Mono octyl = 1.2 Di octyl = 0.04 Di methyl = 0.18 (SML(T) expressed as tin)	Stabilizer for PVC-U
Calcium/zinc stearates	No restriction	Stabilizer for PVC-U and PVC-P
Methylmethacrylate butadiene/styrene	(Polymeric additive)	Impact modifier for PVC-U
Acrylate	(Polymeric additive)	Processing aid for PVC-U
Glycerol monooleate	No restriction	Lubricant in PVC-U
PE wax	(Polymeric additive)	Lubricant in PVC-U
Stearic acid	No restriction	Lubricant in PVC-P
White mineral oil	No restriction	Lubricant in PVC-P
Adipate	Di-2 ethylhexyl adipate = 18 Polymeric = 30	Plasticiser for PVC-P
Epoxidised soya bean oil	No restriction	Plasticiser for PVC-P

**Table 10:** Most common additives in PS manufacturing, describing their functions and specific migration limit (SML) in food or food simulants. Adapted from Table 1 in reference [173].

Monomers and/or Additives	Specific Migration Limit—SML (mg/kg) According to 2002/72/EC	Function
Pentaerythritol tetrakis [3-(3,5-di-tert-butyl-4-hydroxphenyl)propionate]—antioxidant, commercial name Irganox 1010	None	Antioxidant
Octadecyl 3-(3,5-di-tert-butyl-4-hydroxyphenyl)propionate– antioxidant, commercial name Irganox 1076	SML = 6 mg/kg	Antioxidant
White mineral oils	Specification	Processing aids and flow promoters
Zinc stearate	SML = 25 mg/kg, zinc stearate group, expressed as zinc.	Mold release agent

**Table 11:** Adhesives used in food packaging and their more common uses. Adapted from [179], and Annex I in reference [176].

	PVOH	Casein (Natural Polymer)	Starch (Natural Polymer)	Cellulose (Natural Polymer)	PU (Reactive Adhesive)	PVAc (Dispersions/ Emulsions)	Acrylic Polymers and EVA	Coldseals (latex)	Heatseals	Hotmelt Adhesives	Hotmelt PRESSURE Sensitive Adhesive (PSA)
Flexible packaging		**X** Paper to foil laminating		**X** Laminating			**X** Laminating	**X** Sealing	**X** Pharma blister sealing; lidding for dairy products; trays		**X** Reclosable lidding for trays
Folding boxes					**X**	**X**	**X**			**X**	**X**
Three-layer laminates (Substrate 1/Adhesive/Substrate 2)	**X** Paper/paper				**X** PA/PE PET/PE	**X** CB/CB	**X** Paper/PP Paper/PET CB/CB			**X** CB/CB	
Cardboard closing										**X**	**X**
Sacks and bags			**X**		**X**	**X**	**X**		Bag closure	**X**	**X**
Labeling		**X**	**X**						In mold labeling	**X**	**X**
Tissue and towels			**X**	**X**		**X**	**X**				
Sealing packaging								**X** Chocolate bars ice cream	**X** Lidding on aluminum, glass paper pouches	**X**	
Tapes and PSA labels							**X**				**X**

**Table 12:** Ease of recycling the most-used plastics globally [183].

Plastic	Recycling Codes	Ease of Recycling
PET	1	Easy
HDPE	2	Easy
PVC	3	Very difficult
LDPE	4	Feasible
PP	5	Feasible
PS	6	Difficult
Others	7	Very difficult
